# Supplementary material for: Single MVA-SARS-2-ST/N Vaccination Rapidly Protects K18-hACE2 Mice against a Lethal SARS-CoV-2 Challenge Infection
Source: Viruses. 2024 Mar 8;16(3):417. doi: 10.3390/v16030417 (PMC10974247; doi:10.3390/v16030417)
Supplement: Supplementary file 1 [file viruses-16-00417-s001.zip › viruses-2889978-supplementary.pdf]

Supplemental Figures

Supplement to

Single MVA-SARS-2-ST/N vaccination rapidly protects K18-hACE2 mice against a lethal SARS-CoV-2 challenge infection

Supplemental Figures

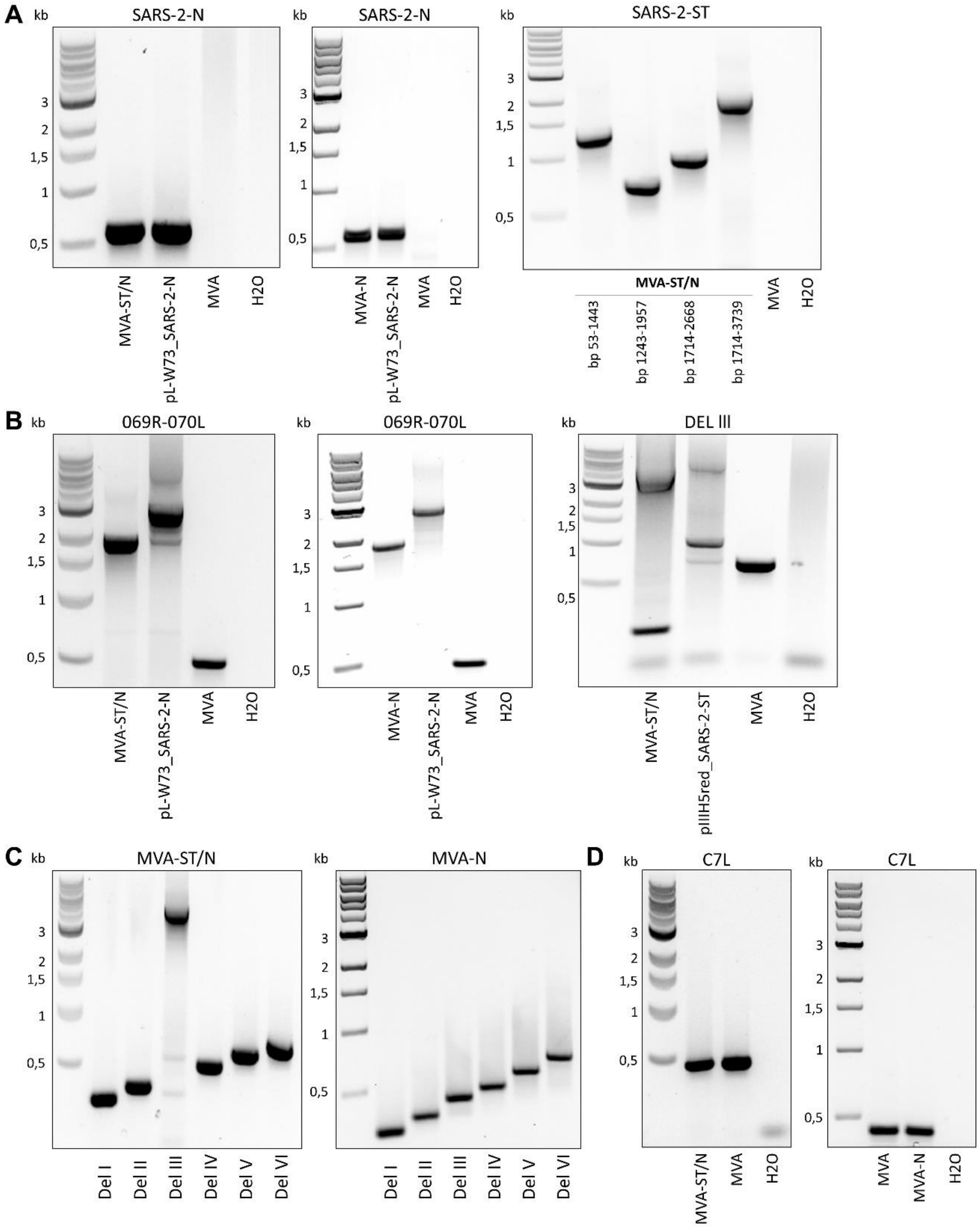

Figure S1: Genetic identity of the recombinant MVA-N and MVA-ST/N candidate vaccines. Genetic identity of MVA-N and MVA-ST/N was confirmed by polymerase chain reaction (PCR) using viral DNA. (A) PCR targeting SARS-2-N and the SARS-2-ST confirming the presence of the SARS-2-N and SARS-2-ST gene sequence with (B) additional PCR demonstrating the site-specific insertion of the sequences as well as the proper removal of the marker genes. (C) Genetic stability was confirmed by PCR targeting the six major deletion sites and (D) the C7L gene locus of MVA.

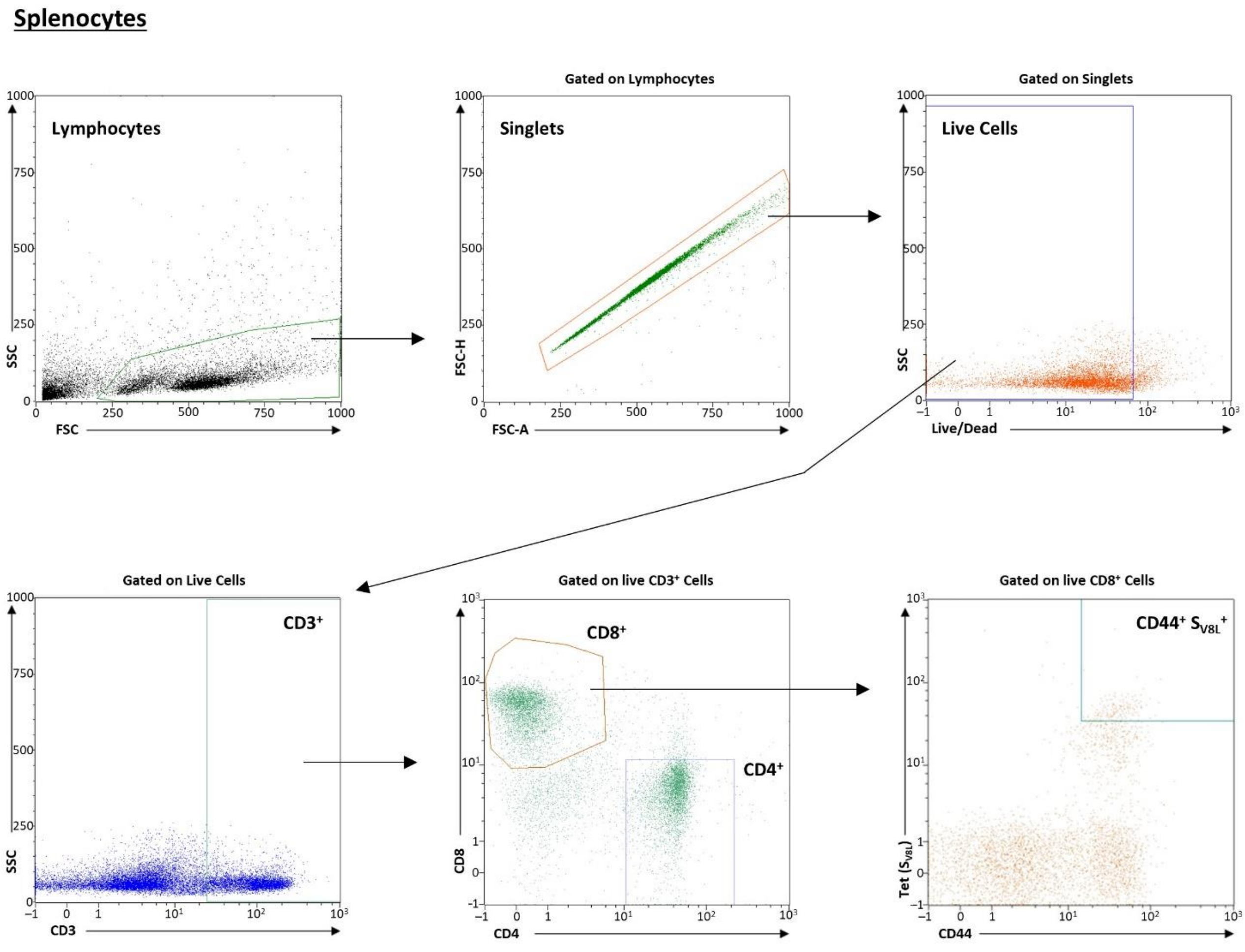

Figure S2: Gating strategy of flow cytometry analysis. The gating strategy for the analysis of splenocytes, lung cells, brain cells and whole blood is shown by splenocytes from an MVA-ST/N vaccinated mouse, which was infected with SARS-CoV-2 28 days post immunization. First, lymphocytes were separated by phenotypical analysis followed by gating on single cells. All live cells

from single cells were gated to depict CD3-positive ($CD3^+$) cells. $CD3^+$ cells were further divided into CD8-positive ($CD8^+$) and CD4-positive ($CD4^+$) cells. Gated on $CD8^+$ cells, T cells specific against the S-protein (S_{V8L}^+) were analyzed by positive tetramer (S_{V8L}) signal and additionally characterized as activated T cells ($CD44^+$). Samples from unvaccinated and unchallenged K18-hACE2 mice, as well as unstained samples from vaccinated and challenged mice, were used to subtract background signaling especially for determining tetramer specific signal. Analysis of N_{219}-specific and $B8R_{20-27}$-specific T cells followed the same gating strategy.

A) SARS-2-N Protein of USA-WA1/2020 (N-Pool)

Peptides 17- to 13-mer, with 10 amino acid overlap

MSDNGPQNQR NAPRITFGGP SDSTGSNQNG ERSGARSKQR RPQGLPNNTA SWFTALTQHG KEDLKFPRGQ GVPINTNSSP DDQIGYYRRA TRRIRGGDGK MKDLSPRWYF YYLGTGPEAG LPYGANKDGI IWVATEGALN TPKDHIGTRN PANNAAIVLQ LPQGTTLPKG FYAEGSRGGS QASSRSSSRS RNSSRNSTPG SSRGTSPARM AGNGGDAALA LLLLDRLNQL ESKMSGKGQQ QQGQTVTKKS AAEASKKPRQ KRTATKAYNV TQAFGRRGPE QTQGNFGDQE LIRQGTDYKH WPQIAQFAPS ASAFFGMSRI GMEVTPSGTW LTYTGAIKLD DKDPNFKDQV ILLNKHIDAY KTFPPTEPKK DKKKKADETQ ALPQRQKKQQ TVTLLPAADL DDFSKQLQQS MSSADSTQA

B) Immunodominant Epitopes of SARS-CoV-2 (USA-WA1/2020)

Single Peptide Sequences

S-Peptide Epitope (S_{V8L})	539-546	(VNFNFNGL)
N-Peptide Epitope (N_{219})	219-227	(LALLLLDRL)
Vaccinia Virus WR Epitope ($B8R_{20-27}$)	20-27	(TSYKFESV)

Figure S3: (A) Protein sequence of SARS-CoV-2 nucleoprotein (N) used for splenocyte and lung cell re-stimulation in ELISpot assay (N Pool). The SARS-CoV-2 nucleoprotein comprises of 419 amino acids. The whole peptide pool, consisting of 59 overlapping peptides, were derived from the SARS-2-N protein sequence. Each single peptide consists of 17 or 13 amino acids (17- or 13-mers) with a 10mer overlap. (B) Single peptide sequences of the immunodominant epitope of the S-protein (S_{V8L}) [1] and the N-protein (N_{219}) [2] of SARS-CoV-2 (USA-WA1/2020) and the immunodominant epitope of MVA ($B8R_{20–27}$) [3]. Single peptides were used for re-stimulation of splenocytes and lung cells in ELISpot assays as well as loaded onto tetramers for the identification of specific T cells within splenocytes, lung cells, brain cells and whole blood by flow cytometry.

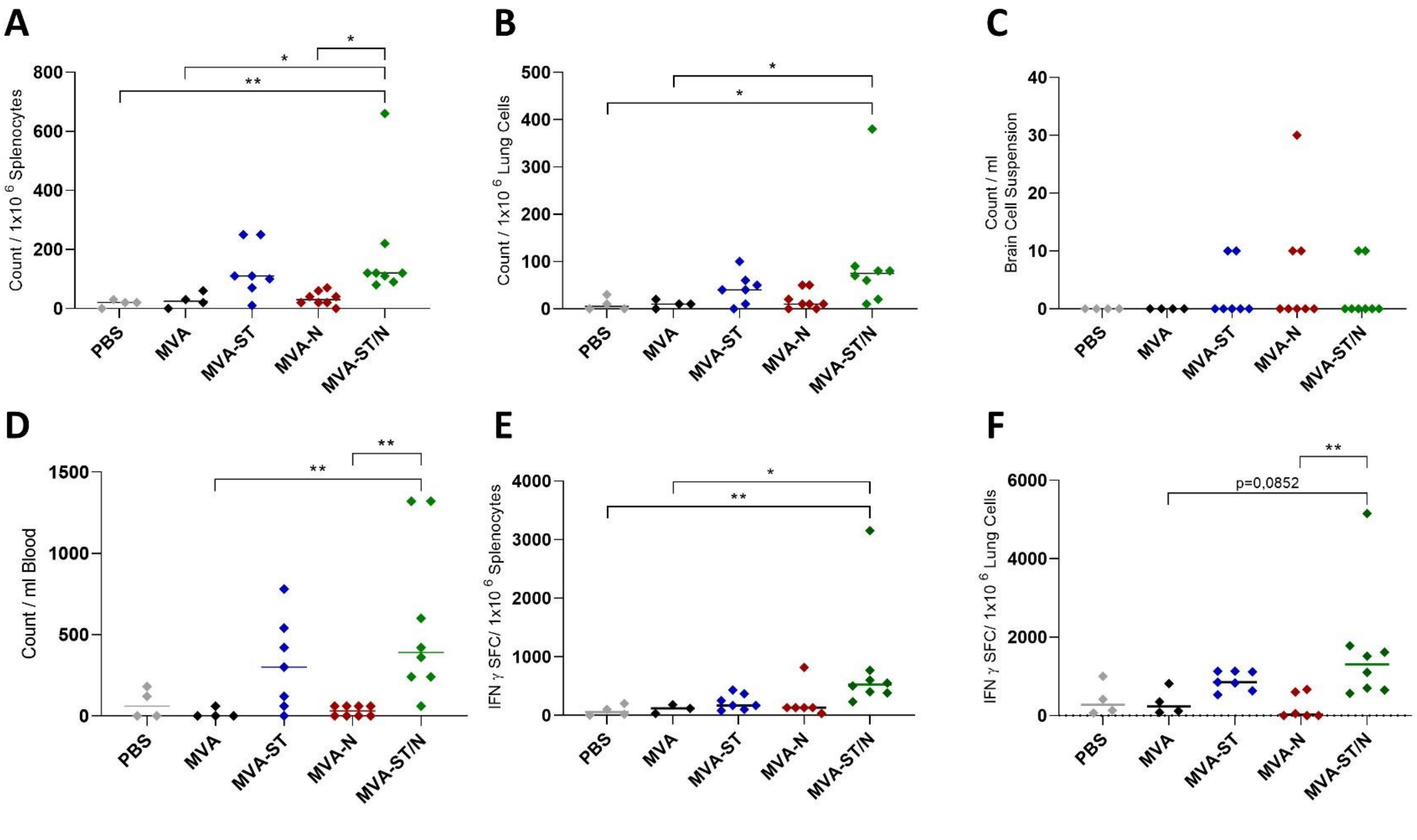

Figure S4: The frequency of expanded MVA_{B8R}-specific $CD8^+$ T cells in spleen, lung, brain, and blood after single shot vaccination. K18-hACE2 mice were challenged with SARS-CoV-2 28 days after intramuscular vaccination with MVA-SARS-2-ST (10^8 PFU, *n* = 7), MVA-SARS-2-N (10^8 PFU, *n* = 8), MVA-SARS-2-ST/N (10^8 PFU, *n* = 8), empty MVA (n = 4) or PBS (*n* = 4). Whole blood was taken

at the day of death and cells were isolated from spleens, lungs, and brains. Splenocytes (A), lung cells (B), brain cells (C) and whole blood samples (D) were analyzed for T cells specific for the MVA epitope $B8R_{20–27}$ using flow cytometry. Splenocytes (E) and lung cells (F) were additionally analyzed for T cells specific for the MVA epitope $B8R_{20–27}$ via ELISpot assay. Differences between the groups were analyzed by Kruskal-Wallis Test. Asterisks represent statistically significant differences between two groups: * $p < 0.05$, ** $p < 0.01$.

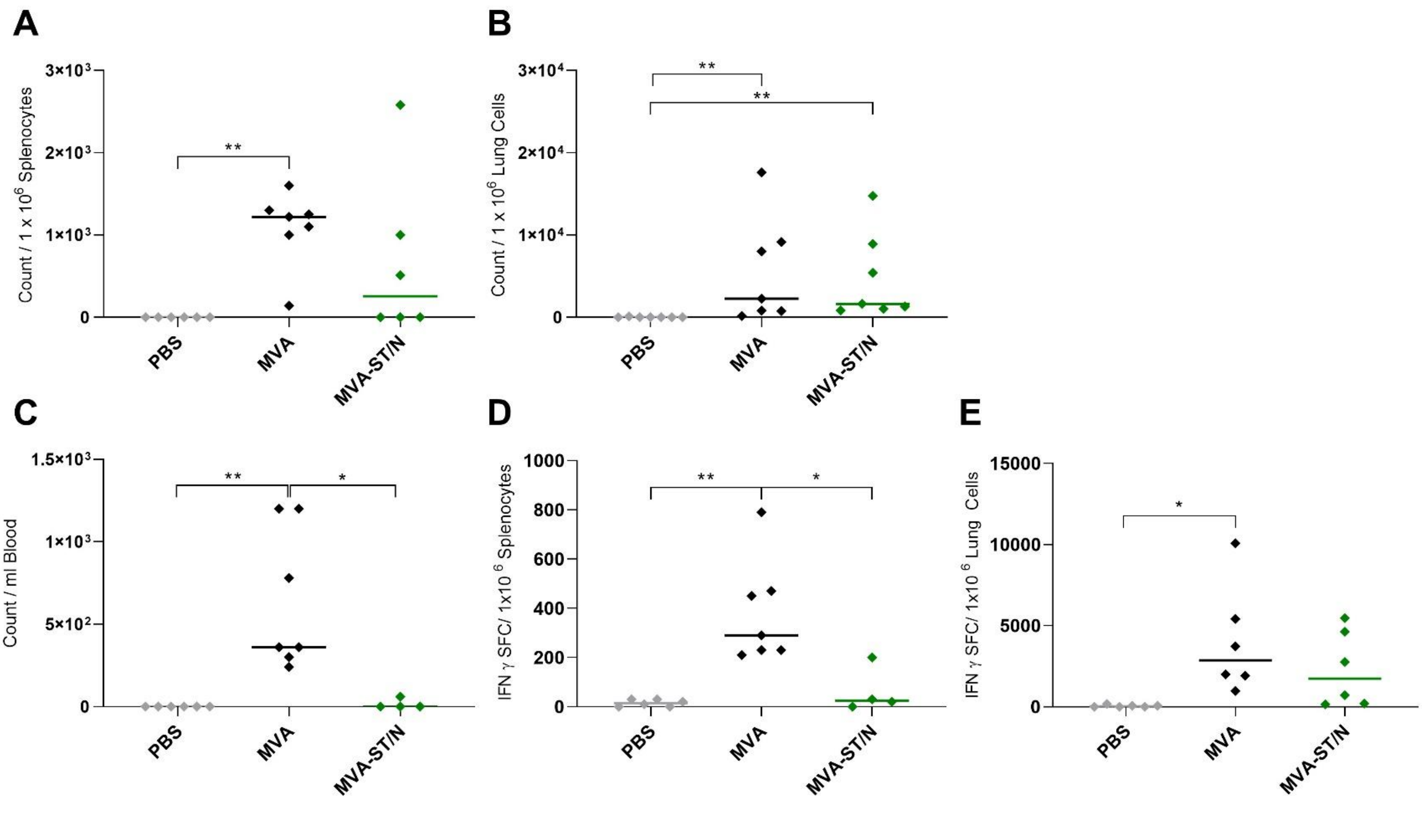

Figure S5: The frequency of expanded MVA_{B8R}-specific $CD8^+$ T cells in spleen, lung, and blood after emergency vaccination. K18-hACE2 mice were challenged with SARS-CoV-2 two days after intramuscular vaccination with MVA-SARS-2-ST/N (10^8 PFU, $n = 7$), empty MVA (n = 7) or PBS ($n = 7$). Whole blood was taken at the day of death and cells were isolated from spleens and lungs. Splenocytes (A), lung cells (B) and whole blood samples (C) were analyzed for T cells specific for the MVA epitope $B8R_{20–27}$ using flow cytometry. Splenocytes (D) and lung cells (E) were additionally analyzed for T cells specific for the MVA epitope $B8R_{20–27}$ via ELISpot assay. Differences between the

groups were analyzed by Kruskal-Wallis Test. Asterisks represent statistically significant differences between two groups: * $p < 0.05$, ** $p < 0.01$.

References

1. Bošnjak, B., et al., *Intranasal Delivery of MVA Vector Vaccine Induces Effective Pulmonary Immunity Against SARS-CoV-2 in Rodents.* Front Immunol, 2021. **12**: p. 772240.
2. Joag, V., et al., *Cutting Edge: Mouse SARS-CoV-2 Epitope Reveals Infection and Vaccine-Elicited CD8 T Cell Responses.* J Immunol, 2021. **206**(5): p. 931-935.
3. Tscharke, D.C., et al., *Identification of poxvirus CD8+ T cell determinants to enable rational design and characterization of smallpox vaccines.* J Exp Med, 2005. **201**(1): p. 95-104.
